# Wastewater based genomic surveillance key to population level monitoring of AmpC/ESBL producing *Escherichia coli*

**DOI:** 10.1038/s41598-025-91516-9

**Published:** 2025-03-03

**Authors:** Ahmad Ibrahim Al-Mustapha, Ananda Tiwari, Riikka Laukkanen-Ninios, Kirsi-Maarit Lehto, Sami Oikarinen, Anssi Lipponen, Tarja Pitkänen, Annamari Heikinheimo, WastPan Study Group, Ananda Tiwari, Kirsi-Maarit Lehto, Sami Oikarinen, Anssi Lipponen, Tarja Pitkänen, Annamari Heikinheimo, Viivi Heljanko, Venla Johansson, Paula Kurittu, Ahmad I. Al-Mustapha, Anna-Maria Hokajärvi, Anniina Sarekoski, Aleksi Kolehmainen, Annika Länsivaara, Rafiqul Hyder, Erja Janhonen, Soile Blomqvist, Kati Räisänen, Carita Savolainen-Kopra, Teemu Möttönen, Oskari Luomala, Aapo Juutinen

**Affiliations:** 1 Department of Food Hygiene and Environmental Health, Faculty of Veterinary Medicine, University of Helsinki, Helsinki, Finland; 2 Department of Veterinary Services, Kwara State Ministry of Agriculture and Rural Development, Kwara State, Ilorin, Nigeria; 3 Department of Veterinary Public Health and Preventive Medicine, Faculty of Veterinary Medicine, University of Ibadan, Ibadan, Oyo State, Nigeria; 4 Department of Public Health, Microbiology Unit, Finnish Institute for Health and Welfare, Kuopio, Finland; 5 Faculty of Medicine and Health Technology, Tampere University, Tampere, Finland; 6 Department of Medicine, Unit of Biomedicine, University of Eastern Finland, Kuopio, Finland; 7 Finnish Food Authority, Ruokavirasto, Seinäjoki, Finland; 8 The Expert Microbiology Unit, Finnish Institute for Health and Welfare, Helsinki, Finland; 9 Infectious Disease Control and Vaccinations Unit, Finnish Institute for Health and Welfare, Helsinki, Finland

**Keywords:** Antimicrobial resistance, Antibiotic resistance genes, Extended spectrum beta lactamase, *Escherichia coli*, One Health, Population surveillance, Epidemiology, Bacteria, Environmental microbiology

## Abstract

Antimicrobial resistance (AMR) is a serious threat to global public health, but obtaining representative data on AMR for healthy human populations is difficult. Here, we leverage the power of whole genome sequencing (WGS) to screen AmpC- and extended-spectrum beta-lactamase (ESBL)-producing *Escherichia coli* from 77 composite samples obtained from 10 wastewater treatment plants across Finland. We found a high abundance of multidrug-resistant AmpC/ESBL-producing *E. coli* and significant differences in the diversity of AMR genes between the sampled cities. The *in silico* analysis of 73 short-read genome sequences shows the clonally diverse isolates consisting of 30 sequence types (STs), including the globally distributed pandemic ST131 clone. The CTX-M ESBL genes were detected in 86.3% (63/73) of the isolates concurrently with the blaTEM-1 (31.5%, 23/73) and blaOXA-1 (9.6%, 7/73) genes. The most prevalent ESBL genes were blaCTX-M-15 (46.6%, 34/73), blaCTX-M-27 (16.4%, 12/73), blaCTX-M-14 (4.1%, 3/73), and blaCTX-M-55 (4.1%, 3/73). Two isolates harboured the carbapenemase resistance gene, blaKPC-2 and blaNDM-1, respectively. In addition, WGS predicted phenotypic resistance against aminoglycosides, beta-lactams, cephalosporins, quinolones, sulfonamides, carbapenems, and polymyxins. The diversity of antibiotic- and stress-resistance genes correlated with the clinical incidence reported in the Finnish AMR report. Core-genome MLST revealed two wastewater genomic clusters but no genomic clusters among human and wastewater ST131 isolates. Our findings suggest the circulation of distinct clonal lineages of AmpC/ESBL-producing *E. coli* across Finland, with variations in AMR gene diversity and abundance by wellbeing service county. Also, our findings underscore the fact that wastewater surveillance could be key to population-level monitoring of AmpC/ESBL-producing *Escherichia coli* and can serve as complementary data to guide public health decisions. We propose longitudinal WGS-based epidemiology as an economically feasible approach for global AMR surveillance, pathogen evolution, and prediction of AMR.

## Introduction

Antimicrobial resistance (AMR) is a cross-cutting and increasing threat to global health, and it threatens to undermine decades of progress in the treatment of infectious diseases^1–3^. The emergence of AMR bacteria is a complex problem with multiple and interconnected drivers, including antibiotic usage, international travel, climate change, and population dynamics – including immigration^2,3^. In most countries, AMR surveillance mainly relies on passive reporting of phenotypic laboratory results for specific pathogens isolated from clinical infections in humans or animals, with limited data from the environment^3–5^. The passive surveillance from clinical isolates are typically targeted towards priority multidrug-resistant (MDR) pathogens. Moreover, it is usually associated with significant time delays, a narrow pathogen spectrum, and is only feasible in patients who present themselves to health facilities^4,5^.

In the EU, human AMR surveillance relies on passive reporting of clinical infections. Hence, the true AMR carriage in the population is not known. Therefore, wastewater offers a possible screening point for AMR carriage in human populations^2,6–8^. Across the globe, wastewater-based surveillance (WBS) became prominent in population-level monitoring of SARS-CoV-2 variants^2,9,10^, in addition to its use in monitoring other priority pathogens such as polioviruses^11^, Influenza viruses^12^, and illegal drug use^13^. For AMR, several collaborative efforts use WBS as a valuable tool in monitoring AMR carriage trends and their environmental load^14–20^. In addition, there is a new EU regulation that mandates monitoring AMR in wastewater treatment plants (WWTPs) and recognizes it as an invaluable tool for the genomic epidemiology of pathogens^21,22^. For global uniformity, the tripartite organization (World Health Organization, World Organization for Animal Health and the Food and Agriculture Organization) agreed to use the trends of the extended-spectrum beta-lactamases (ESBL)-producing *Escherichia coli* as a global indicator to monitor the success in curbing the emergence and spread of AMR bacteria^23^. This organism was proposed because of its increasing zoonotic potential, its potential to transfer resistance determinants to other gut bacteria, and its ease of culture – especially in resource-poor settings in Africa and Asia^23^.

In Finland, the burden of AmpC/ESBL-producing *E. coli* was reported to be about 6% between 2012 and 2019; and a prevalence of 5-7% were reported to the infectious disease register in 2021^24^. Three-quarters of these cases were isolated from women, half of total cases were in patients over 62 years old, and 52% were cultured from urine samples^24,25^.

Advancements in genomic technologies like WGS, as well as in bioinformatics have revolutionized the study of AMR by allowing comprehensive analysis of antimicrobial resistance genes (ARGs) and mobile genetic elements in wastewater^2,5^. This study used WGS of single bacterial colonies cultured from untreated sewage from ten WWTPs to characterize the diversity of resistance determinants in AmpC/ESBL-producing *E. coli*. The findings of this study are expected to enhance the understanding of the genomic characteristics of AmpC/ESBL-producing *E. coli* in municipal wastewater. This knowledge can be instrumental in assessing the diversity of AmpC/ESBL-producing *E. coli* strains circulating in Finland, along with assessing public health risks and developing targeted interventions to control the spread of AMR.

## Materials and methods

### WWTP data, study settings, and sampling

A total of 77 composite wastewater samples (1 litre each) were collected from influents at ten wastewater treatment plants (WWTPs) across Finland (Supplementary Figure S1) between February 2021 and January 2022, as a part of the WASTPAN project^9,26^. These WWTPs were selected to represent the 24 wellbeing service counties in Finland that cater to two-thirds of the Finnish population^27^. The samples were aseptically collected and immediately transported by ice packs to the bacteriology Laboratory of the Department of Food Hygiene and Environmental Health at the University of Helsinki for further analysis.

### Isolation of AmpC/ESBL-producing Escherichia coli isolates

AmpC/ESBL-producing *E. coli* were culturally isolated by the direct plating of 100μl of composite wastewater on CHROMagar ESBL agar plates (CHROMagar orientation + ESBL-supplement) (Paris, France), a clinical chromogenic medium, and incubated for 18–24 hours at 37˚C. From each plate, one presumptive ESBL-producing *E. coli* colony (pink/red) was sub-cultured on fresh CHROMagar plates and finally purified on nutrient agar (Oxoid, Basingstoke, UK). Bacterial species were identified using the Matrix-Assisted Laser Desorption Ionization Time of Flight - Mass Spectrometry (MALDI-ToF) (Bruker, Bremen, Germany) following the Biotyper protocol using the best match score value of ≥ 2.300.

### Phenotypic ESBL production assay

The double-disk synergy test (DDST) was used to test for the production of extended-spectrum beta-lactamases in all confirmed *E. coli* isolates (n = 75) as previously described^28^. Briefly, an isolate was regarded as an ESBL producer if it showed resistance towards third-generation cephalosporins [cefotaxime - 30µg] and the difference in the zone of inhibition between the cefotaxime (30µg) and cefotaxime + clavulanate (30µg + 10µg) was more than 5mm. In addition, we screened for resistance against fluoroquinolones using the ciprofloxacin 10µg disk. All antibiotic disks (Neo-Sensitabs) were sourced from Rosco Diagnostics, Denmark. All measurements and interpretations were based on the epidemiological cut-off (ECOFF) values established by the European Committee on Antimicrobial Susceptibility Testing guidelines^29^. The reference strain*, E. coli* ATCC 25922, was used as the negative control strain during all screening tests. A graphical presentation of the methodology is presented in Supplementary Figure S2.

### Whole genome sequencing of AmpC/ESBL-producing E. coli

The genomic DNA of the isolates was extracted using the DNeasy blood and tissue Kit (Qiagen, Hilden, Germany) in a QIACUBE Connect (Qiagen, Hilden, Germany) following the manufacturer’s instructions. The purified DNA was quantified with a Qubit Fluorometer 4.0 (Invitrogen, Singapore). DNA sequencing was outsourced from a commercial sequencing service provider (Novogene GmbH, Munich, Germany) which used the Illumina PE150 technology for microbial whole genome sequencing. The raw sequencing read output had its adaptor trimmed using Trimmomatic (*v. 0.36*), and the quality of the reads was assessed using the FastQC tool *v. 0.11.9* using a threshold of 90% as required identity to reference sequence and 99% as required percentage alignment with reference genome respectively. The raw reads were assembled into contigs using Skesa *v. 2.4.0*.

The sequences have been deposited at the European Nucleotide Archive (BioProject PRJEB78480) with the individual accession numbers given in Supplementary File 1. The assembled contigs were fed into the RIDOM SEQSPHERE+ *v. 10.0* (Ridom GmbH, Münster, Germany). This bacterial analytical pipeline utilized the NCBI AMR Finder plus tool (*v. 3.11.26*) with the default threshold of 90% identity and 60% minimum length to identify resistance genes. The presence of class 1 integrons was screened using Integrall *v. 1.2*. We screened for IS*26,* an insertion sequence element that has been reported to be prevalent in environmental samples using the online Blast tool of ISFinder *v.2.0*. Afterwards, we screened for the transmissible locus of stress tolerance (tLST) variants by blasting the tLST variants (tLST1 and tLST2) against the 73 ESBL-*E. coli* genomes using Blastn (https://blast.ncbi.nlm.nih.gov/BlastAlign.cgi). The tLST ORFs screened were obtained from Zhang and Yang^30^. The CGE analytical pipeline was used to identify the plasmid replicons (PlasmidFinder *v.2.1*) and serotypes (SeroTypeFinder *v.*2.0) using the recommended settings. Finally, the isolates were classified into phylogroups using the Clermont Phylotyper tool *v*.*1.4.0.* We conducted the core genome Multi-Locus Sequence Typing (cgMLST) against 2513 targets for gene-by-gene allele calling to investigate the genetic relatedness of the assembled genomes as previously described^31^. A cluster alert distance of 10 allele differences and a cluster alert quality threshold of at least 95% good cgMLST targets were used to detect closely related isolates. We further compared the wastewater ST131 isolates (n = 18) with human ST131 isolates (n = 21) reported by Kurittu et al.^28^ to identify genomic clusters of ST131 infections. A cut-off of 10 allelic differences was used to establish relatedness (clusters) among isolates.

### Data analysis

All statistical analysis was done in SPSS *v.28.* The chi-square analysis was used to test for associations between the relative abundance of plasmids and ARGs in *E. coli* isolates that were obtained from the ten WWTPs. The Sankey diagram was computed in Power BI^®^ (Microsoft Incorporation, USA). The ARG diversity map was generated using the ‘geocoding’ function in Ridom SeqSphere+.

## Results

### Occurrence of ESBL producing Escherichia coli in wastewater

Cultivation of wastewater using the ChromAgar ESBL revealed that 97.5% (n = 75) of the 77 composite wastewater samples contained tentative AmpC/ESBL-producing *E. coli*. The double-disk synergy test revealed that 86.7% (n = 65/75) of the isolates were true ESBL-producing isolates (Supplementary file 2). Phenotypic resistance to ciprofloxacin was expressed by 82.7% (n = 62/75) of the isolates. Spatially, AmpC/ESBL-producing *E. coli* was detected in the ten WWTPs and from all sampling periods (February 2021 – January 2022) across Finland. All 75 isolates were sequenced, but two isolates did not pass the quality control and were exempted from further analysis.

### In silico genotyping of AmpC/ESBL-producing Escherichia coli in wastewater

The 73 sequenced isolates belonged to phylogroup A (n = 15), B1 (n = 3), B2 (n = 31), C (n = 2), D (n = 16), and F (n = 6). The isolates belonged to 30 different multi-locus sequence types (STs) (Supplementary File 3). Eighteen isolates (24.7%) belonged to the globally distributed ST131, seven (11%) isolates belonged to ST69, and four (5.5%) isolates belonged to ST1193. The other isolates (n = 44) belonged to several sequence types (Supplementary File 3). There were significant differences in the presence of class 1 integrons (*intI1* gene), the number of plasmid replicons, as well as the diversity and abundance of antibiotic, disinfectant, and stress-resistance genes across the phylogroups (p < 0.05). Our dataset further revealed that 43.8% (n = 32/73) of the isolates harboured the *IntI1* gene, especially among isolates belonging to phylogroup A (Table 1). The IS*26* element was detected in most (91.8%, n=67/73) of the genomes screened. In terms of plasmid replicons, all three isolates belonging to phylogroup B1 had the highest number of plasmid replicons, whereas those belonging to phylogroup B2 had the least number of plasmid replicons (41.9%, n = 13/31). The *qacE/qacEΔ1/qacL* gene was not detected in any of the five isolates belonging to phylogroup B1 (n = 3) or phylogroup C (n = 2). The two tLST variants (tLST1 and tLST2) were detected in 5.5% (n=4) of the 73 genomes. The four isolates harboured both tLST1 variants and tLST1 had complete ORFs and a few ORFs were missing in the tLST2 (Supplementary File 4).

**Table 1.** Distribution of genomic features among diverse phylogroups of AmpC/ESBL-producing *E. coli* (n = 73).

		*IntI*1	tLST	IS*26*	Plasmid replicons (1 or more)	Point mutations (1 or more)	3 or more ARGs	*qac*E*/qacEΔ1*	*ymg*B
Phylogroups	A (n = 15)	11 (73.3)	3 (20)	15 (100)	11 (73.3)	15 (100)	14 (93.3)	9 (60)	13 (86.7)
	B1 (n = 3)	0 (0)	0 (0)	3 (100)	3 (100)	1 (33.3)	2 (66.7)	0 (0)	3 (100)
	B2 (n = 31)	13 (41.9)	1 (3.2)	29 (93.5)	13 (41.9)	31 (100)	18 (58.1)	8 (25.8)	31 (100)
	C (n = 2)	0 (0)	0 (0)	1 (50)	1 (50)	1 (50)	0 (0)	0 (0)	2 (100)
	D (n = 16)	4 (25)	0 (0)	14 (87.5)	11 (68.8)	6 (37.5)	8 (50)	4 (25)	16 (100)
	F (n = 6)	4 (66.7)	0 (0)	5 (83.3)	4 (66.7)	5 (83.3)	6 (100)	2 (33.3)	6 (100)

### Serotypes

Analysis of the 73 isolates revealed a high molecular diversity of the isolates, with most isolates belonging to serotype O25:H4 (15%, n = 11/73) and O16:H5 (8.2%, n = 6/73). For ten isolates, the O antigen could not be assigned, and the H antigen could not be assigned for two isolates (Table 2).

**Table 2.** *In silico* serotyping of AmpC/ESBL *Escherichia coli* isolates from wastewater based on whole genome sequencing serotyping (n = 73).

O antigen	H antigen	No. of genomes
O1	H6(1), H25(2), H15 (1), H7 (1)	5
O2	H6 (2)	2
O7	H18 (1)	1
O8	H21 (1), H30 (1)	2
O9	H30 (1), H34 (1)	2
O11	H6 (1), H52 (1)	2
O15	H6 (1), H18 (1)	2
O16	H5 (6)	6
O18ab	H55 (1)	1
O19	H12 (1)	1
O21	H7 (1)	1
O25	H4 (11)	11
O29	H10 (1)	1
O45	H6 (1)	1
O64	H4 (1)	1
O75	H7 (1)H5 (5)	6
O83	H1 (1), H4 (1), H42 (1)	3
O84	H7 (1)	1
O86	H18 (3)	3
O102	H6 (3)	3
O107	H5 (1)	1
O130	H26 (2)	2
O166	H15 (1),	1
O12, O25	-	2
Others	:H4 (2), -:H9 (2), -:H10 (1), -:H16 (1), -:H17 (1), -:H18 (4), -:H25 (1)	10

### Diversity of resistance determinants in AmpC/ESBL-producing Escherichia coli

The CTX-M ESBL genes were detected in 86.3% (n = 63/73) of the isolates concurrently with the *bla*_TEM-1_ (31.5%, n = 23/73), and *bla*_OXA-1_ (9.6%, n = 7/73) genes that confer AmpC resistance. Two isolates (AE22 and AE71) harboured only the AmpC gene, whereas eight isolates were non-AmpC/ESBL isolates that harboured other resistance mechanisms. The ESBL genes detected were *bla*_CTX-M-15_ (46.6%, n = 34/73), *bla*_CTX-M-27_ (16.4%, n = 12/73), and three isolates each harboured the *bla*_CTX-M-14_ and *bla*_CTX-M-55_. Two isolates (AE02 and AE33) harboured the carbapenemase resistance gene *bla*_KPC-2_ and *bla*_NDM-1_ gene, respectively. The *bla*_CTX-M-1,_ *bla*_CTX-M-2,_ and *bla*_SHV-12_ were each detected in a single isolate. Other less abundant ESBL genes can be found in Supplementary File 5.

Other clinically important antibiotic resistance determinants detected in the isolates include *aac*(6’)-*Ib-cr5* (16.4%, n = 12/73) and *aad*A1 (9.6%, n = 7/73). Moreover, a combination of *aad*A5 / *aph*(3’’)-Ib / *aph*(6)-Id was detected in 11% (n = 8/73) of the isolates, and fluoroquinolone resistance determinants were detected in 82.2% (n = 60/73) of the isolates. The plasmid-mediated quinolone resistance gene, *qnr*S1, was detected in 23.3% (n = 17/73) of the isolates, whereas chromosomal point mutations that could result in fluoroquinolone resistance were detected in 80% of the isolates (n = 59/73). These mutations include the *gyrA*_S83L (50.7%), *gyrA*_D87N (35.6%, n = 26/73), *parC*_S80I (37%, n = 27/73), *parC*_E84V (15%, n = 11/73), and the *parE*_I529L (21.9%, n = 16/73). Other ARGs detected include the *sul*1 (28.8%, n = 21/73), *sul*2 (31.5%, n = 23/73), *tet*(A) (26%, n = 19/73), *tet*(B) (13.7%, n = 10/73), *dfr*A14 (12.3%, n = 9/73), *dfr*A17 (20.5%, n = 15/73), and *mph*A genes (30.1%, n = 22/73). The *mcr*-*1.1* that confers resistance to a last resort antibiotic – colistin – was detected in only one isolate (AE59). Other point mutations that could confer resistance to chloramphenicol and rifampin [*marR*_S3N (12.4%, n = 9/73)], and fosfomycin [*uhpT*_E350Q (16.4%, n = 12/73), and *ptsI*_V25I / *uhpT*_E350Q (24.7%, n = 18/73)].

**Fig. 1:**
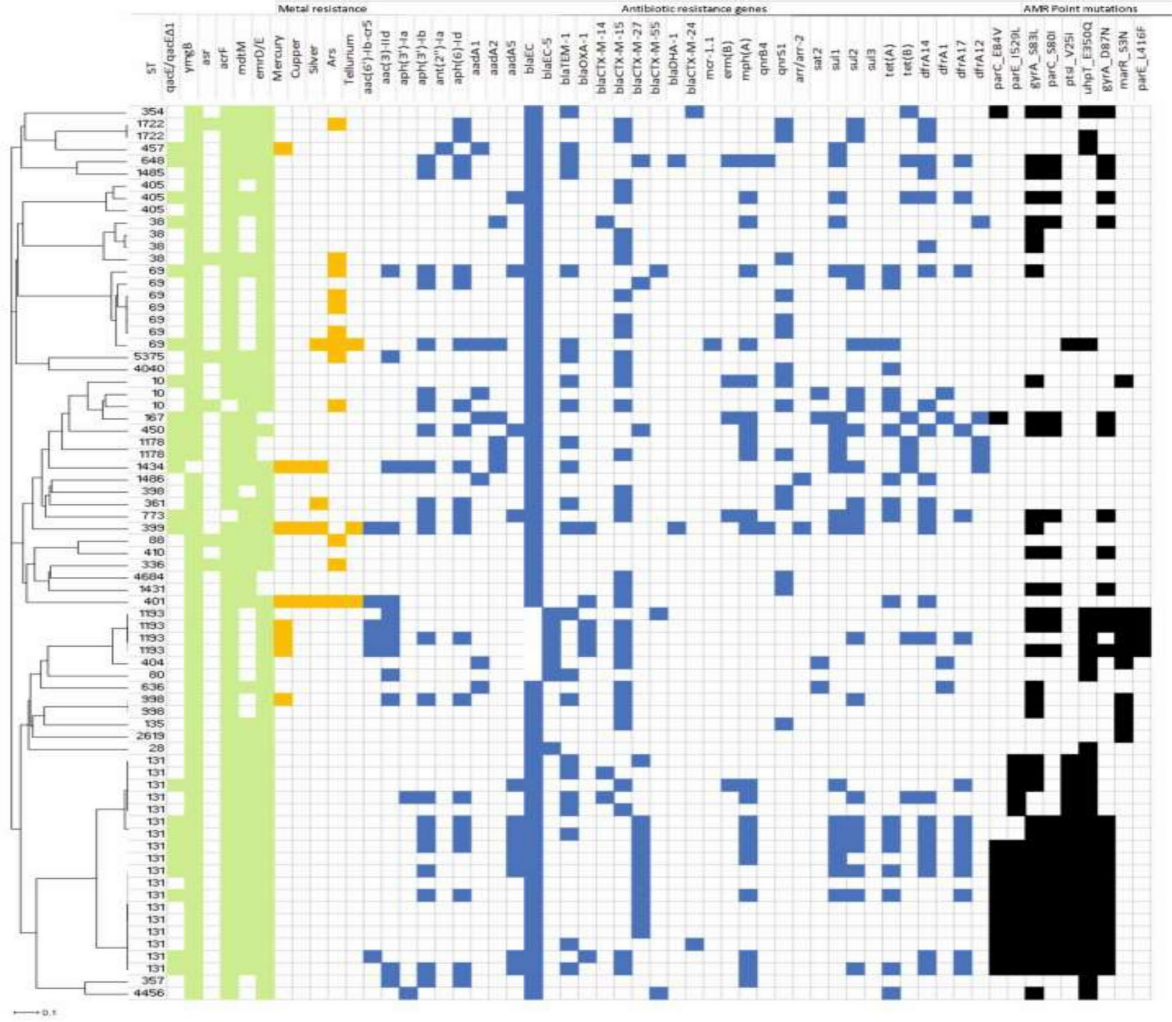
The presence of antibiotic, disinfectant, and stress resistance genes in AmpC/ESBL-producing *Escherichia coli* isolates from wastewater predicted by *in silico* genotyping. Green boxes – efflux pumps; Yellow boxes - metal resistance genes; Blue boxes – antibiotics resistance genes; and Black boxes indicate chromosomal mutations that confer antimicrobial resistance.

Several genes related to stress, disinfectant, heavy metals, and antibiotic resistance were detected in the isolates. Most of the isolates (97.3%, n = 71/73) harboured the *ymgB*, a biocide/acid resistance gene known to be involved in acid resistance and regulating biofilm formation. The disinfectant resistance gene, *qacE/qacEΔ1* gene was detected in 21 isolates, and a single isolate (AE59) harboured the *qacL* gene (Figure 1). There was a high abundance of multidrug efflux pump genes such as *acrF* (97.3%, n = 71/73), *emrD/E* gene (79.4%, n = 58/73), and *mdtM* (71.2%, n = 52/73). In addition, the acid shock protein gene (*asr*) was detected in six (8.2%) of the isolates. These six isolates (AE04, AE06, AE08, AE09, AE24, and AE25) also harboured genes that confer resistance to arsenic (*ars*C, *ars*D, *arsR*), a heavy metal. Other heavy metal genes detected include those that confer resistance to mercury (11%, n = 8/73), silver (6.8%, n = 5/73), copper (4.1%, n = 3/73), and tellurium (4.1%, n = 3/73). While most of the isolates harboured one or two of these heavy metal resistance genes, AE50 and AE54 harboured resistance to mercury, copper, silver, and tellurium. The function/classification of genes associated with antibiotic resistance is provided in Supplementary Table S1.

### Spatial variations in acquired resistance genes

**Fig. 2:**
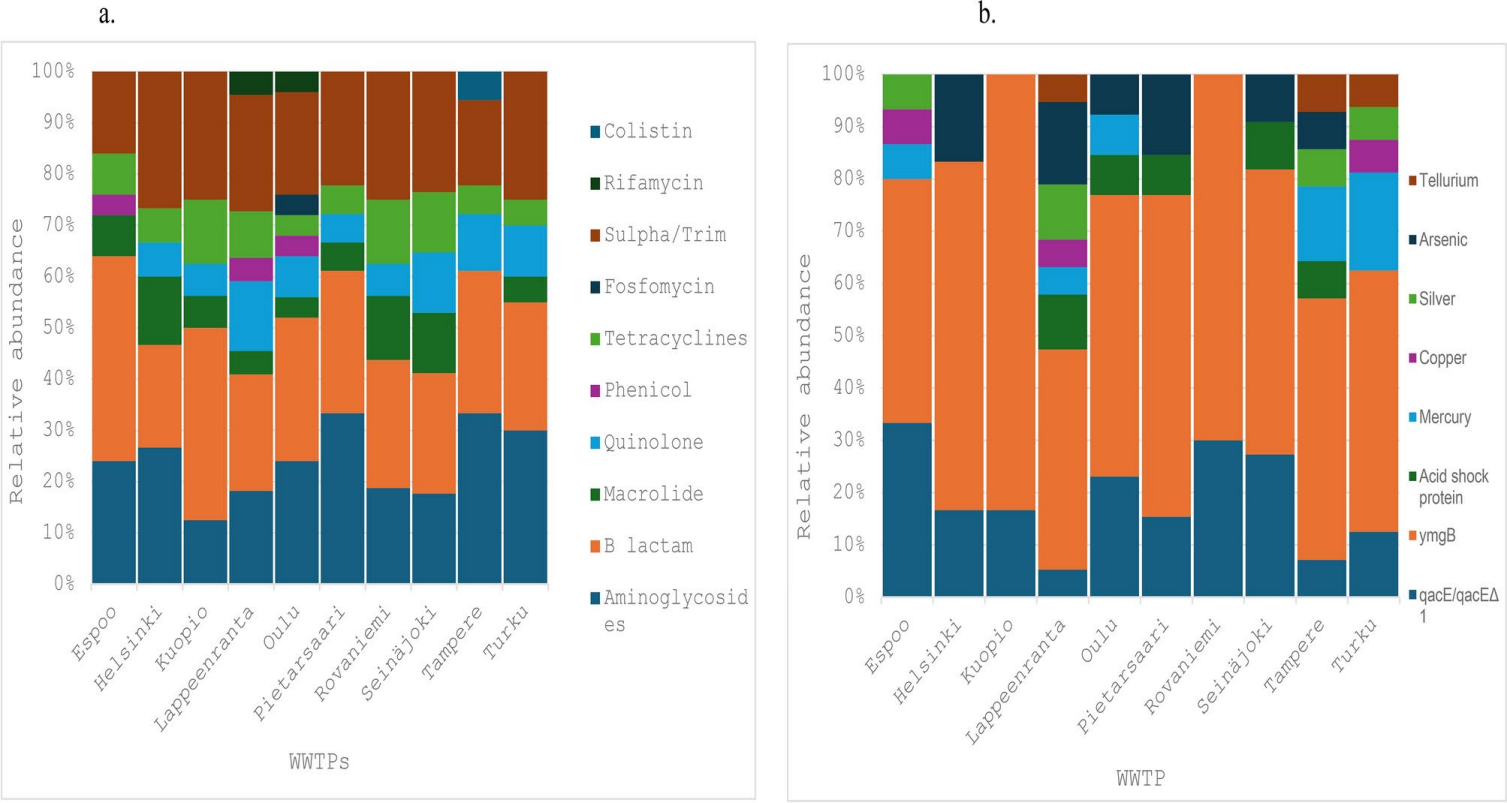
(**a**) Relative abundance of antibiotic resistance genes per antimicrobial class across the 10 major wastewater treatment plants (WWTPs) in Finland. (Sulfa/Trim - Sulfamethoxazole/Trimethoprim). (**b**) Relative abundance of the metal and stress resistance genes across the sampled WWTPs.

The *in silico* analysis revealed that there were more ARGs detected in samples from Oulu and Lappeenranta, although there were no significant differences in the diversity of acquired antimicrobial resistance genes across the other WWTPs (Fig. 2a). However, there were significant differences in the diversity and abundance of the stress resistance genes, with Kuopio and Rovaniemi having the least diversity (only *ymg*B and *qac*E/*qac*EΔ1). Lappeenranta had the highest diversity of stress resistance genes with, isolates from these cities harbouring resistance determinants such as those that confer resistance to copper (*pcoE, pcoS,pcoR, pcoD. pcoC, pcoB, pcoA*), mercury (*merC, merT, merR, merP*), silver (*silE, silP*), arsenic (*arsC, arsD, arsR*), and tellurium (*terD, terW, terZ*), in addition to *asr*, *ymg*B, and *qac*E (Fig. 2b). There were also significant differences in plasmid diversity and relative abundance (p < 0.05) across the WWTPs, with an average of 1.8 plasmid replicons per bacterial isolate in samples from Lappeenranta, compared to the 3.6 plasmids per isolate from Espoo (Supplementary Figure S3). There was no association between the presence of plasmid replicons and the diversity of ARGs in wastewater samples from each WWTP (p = 0.307).

### Genomic diversity of AmpC/ESBL-producing Escherichia coli

**Fig. 3:**
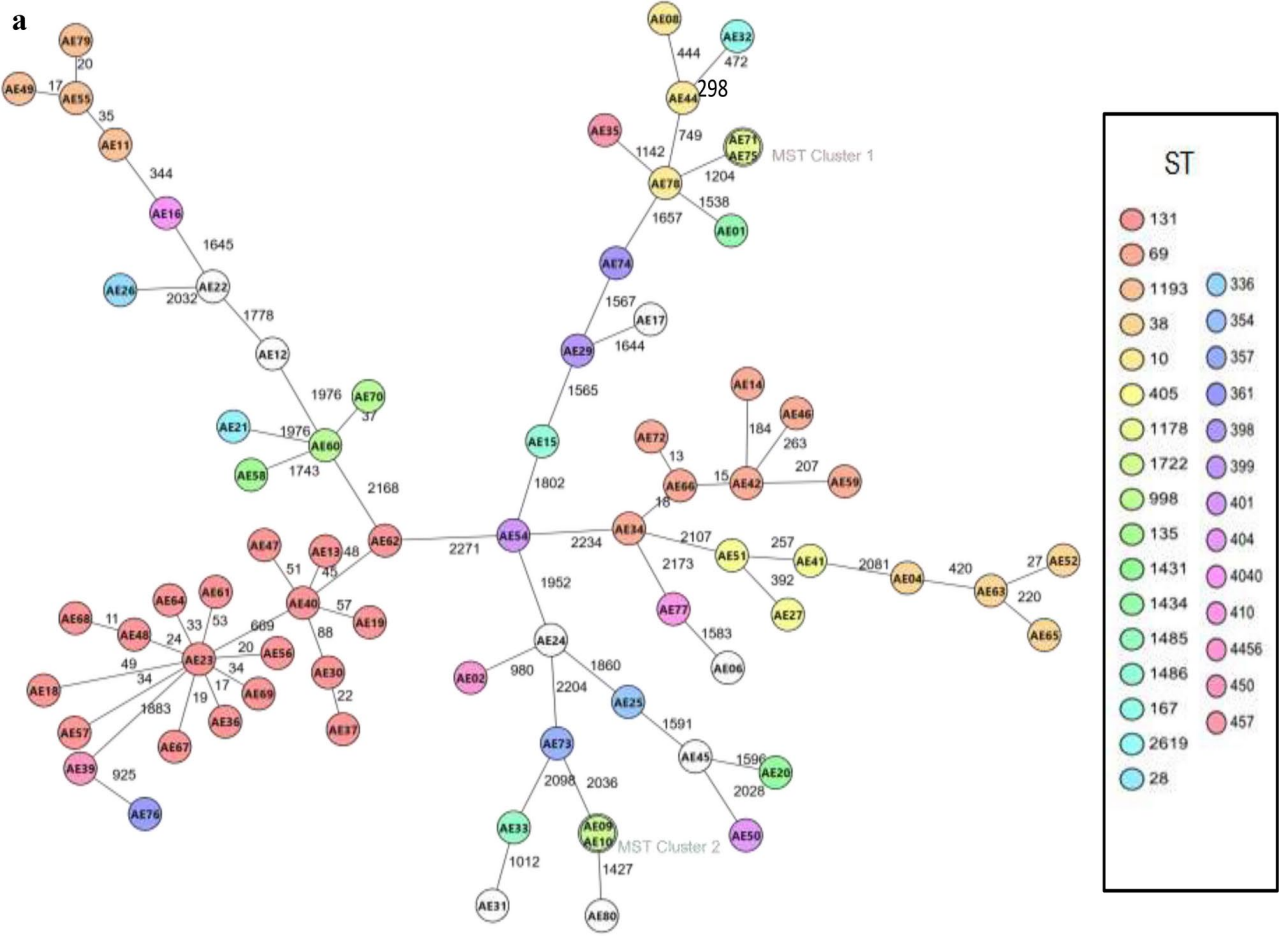

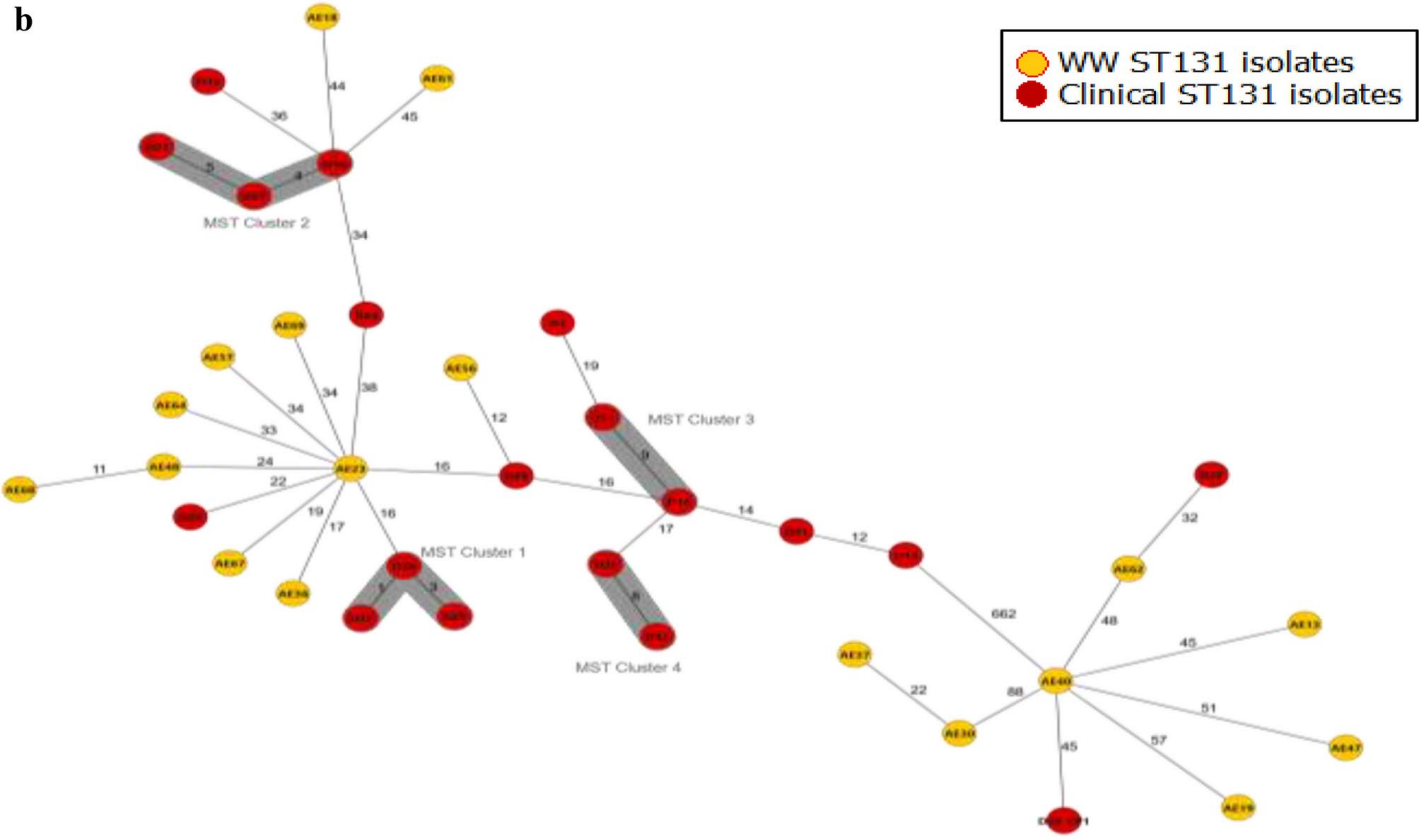
(**a**) Minimum Spanning Tree showing relatedness of wastewater ESBL-producing *E. coli* isolates (n = 73). The Ridom SeqSphere+ MST was based on 2513 genes using a cluster distance threshold of 20. (**b**) Minimum Spanning Tree from the core genome MLST of 39 ST131 isolates from wastewater isolates (n = 18) from this study and human isolates (n = 21) from Kurittu et al.^28^ in Finland. WW- wastewater. The Ridom SeqSphere+ MST was based on 2513 genes using a cluster distance threshold of 10.

The cgMLST revealed high genomic diversity among the 73 AmpC/ESBL with only two genomic clusters (Fig. 3a). The ST131 isolates (n=18), ST 69 (n=7) as well as the four isolates each that were of ST38 and ST1193 all revealed high genomic diversity (Fig. 3a; Supplementary File 3). Samples that made up the first of the two genomic clusters were collected during the same month but from different cities (Espoo: AE71 - A/ST1178/O130:H26-*bla*_CTX-M-15_ and Oulu: AE75 - A/ST1178/O130:H26-*bla*_TEM-1_). The second MST cluster was obtained in February of 2021 from two cities in Western Finland (Tampere and Turku) and genotypically characterized as phylogroup F/ST1722/O1:H25-*bla*_CTX-M-15_. The minimum spanning tree (MST) generated from cgMLST of the 39 ST131 isolates revealed four clusters from the human ST131 isolates. There was however no genomic cluster between human and wastewater isolates buttressing the hyperclonality of the ST131 sublineages (Fig. 3b). There were at least 12 allelic differences in the core genomes of the closest isolates between the human and wastewater isolates (D29 and AE56) with both isolates typed as phylogroup B2/ST131/O25:H4-*bla*_CTX-M-27_.

## Discussion

Longitudinal wastewater-based genomic epidemiology of AMR is crucial to its utility as a tool to evaluate the introduction and burden of priority AMR pathogens. In addition, it could help determine the effects of interventions, identify national and global priorities and identify areas for further research^7,8,32^. From a surveillance point of view, urban wastewater is attractive because it provides sampling material from a large and mostly healthy population, which otherwise would not be feasible to monitor^7,8^. Through the WASTPAN project, we have previously identified urban sewage as a reliable tool for monitoring pathogens^12,26,33–36^ and supporting other surveillance activities^3,37,38^.

The presence of AmpC/ESBL-producing *E. coli* in all WWTPs could be attributed to the ubiquitous nature of *E. coli* as well as the increasing trends in the proportion of ESBL-producing *E. coli* among human *E. coli* isolates in Finland^25^ and across the world^3,38,39^. In addition, international travel, especially to high-prevalence countries, is associated with the acquisition of ESBL-producing *Enterobacteriaceae*^40,41^ and the development of community-onset ESBL-producing *E. coli* infections^25,42^. Another study revealed that 20% of Finnish travellers who had visited destinations outside the Nordic countries have become colonized by ESBL-producing *E. coli*^43^, and the probability of onward transmission of ESBL-producing *Enterobacteriaceae* within households of travellers has been estimated to be 12%^44^.

The phenotypic resistance to ciprofloxacin expressed by 82.7% of our isolates aligned with the 70 - 80% ciprofloxacin resistance expressed among the human samples (urine and blood) screened in Finland between 2008 and 2019^25^. Several studies have shown that antimicrobial use (AMU) selects for certain AMR determinants^45,46^. It has also been suggested that AMU explains only some of the variation^41^ and that other factors such as diet, cultural traditions and occupation also have an influence^47^. In general, the antibiotic consumption rate per antibiotic class (Supplementary Figure S4) was associated with the antibiotic resistance patterns detected in our isolates. However, the occurrence of fluoroquinolone resistance was high despite the low prescription rate of fluoroquinolones in Finland (22/1000 adults/year)^48^. The MDR AmpC/ESBL-producing *E. coli* clinical isolates have been recently reported in Finland^25,28^. This could be because genes encoding for ESBL production are often co-located on the same mobile genetic element that carries genes conferring resistance to fluoroquinolones and aminoglycosides^25,28,45,46,49^.

The sequenced isolates were diverse, with the pandemic O25:H4-ST131- *bla*_CTX-M-15_ and O16:H5-ST131- *bla*_CTX-M-27_ belonging to phylogroup B2 being the most common. These pandemic strains (from the same sublineage) have been previously reported as an emerging intercontinental strain^50,51^, and have been characterized by an extensive virulence profile from both hospital and community isolates of several countries^52,53^ and in day-care centres in France^54^. These strains were also the most prevalent in clinical isolates from Finland^28^. This emerging strain was later detected in companion animals across Europe^55^. Four isolates belonged to the O75:H5-ST1193 strain, an emerging global multidrug high-risk clone^56,57^. This strain is an important cause of community-onset urinary and bloodstream infections^56–58^. These suggest that urban sewage could be a useful complementary tool to detect prevalent clones of AMR pathogens^32,37^.

The diversity of the isolates was further depicted by the wide variety of resistance determinants. The *ymg*B and *asr* genes were previously demonstrated to encode a protein important for the bacterial acid-resistance phenotype^59^, and regulation of biofilm formation. Furthermore, the genes were reported to be involved in an interplay of multifaceted stress responses in *E. coli* on exposure to glutathione and ciprofloxacin^60^. In agreement with the finding of Sutterlin et al.,^61^ screening of urine samples from Sweden, Germany, and Spain detected the chromosomal *ars* operon (arsenic resistance) that all belonged to non-B2 phylogenetic groups. Studies have shown that the occurrence of heavy metal resistance genes (HMRGs) was significantly associated with the presence of disinfectant- or antibiotic-resistance genes^62,63^. However, more research is needed to fully understand the molecular promoters of high occurrence of HMRGs in two isolates (AE50 and AE54) that have four different HMRGs. The prevalence of the tLST variants showed were higher than the 2%^64^ and 2.6%^30^ prevalence previously reported. Our findings also corroborated the findings of these researchers that the tLST ORFs were more prevalent in isolates belonging to phylogroup A. In addition, there was a negative correlation between the presence of ARGs and tLST ORFs among phylogroup A isolates with more ARGs detected in tLST-negative isolates^30^. Finally, we observed that tLST-positive genomes also harboured resistant determinants against heavy metals, especially mercury, copper, and silver.

Our data further supports the evolving epidemiology and diversity of beta-lactamase genes^2,7,65,66^. This study recorded a higher prevalence of the *bla*_CTX–M–15_ gene, unlike the findings of Kurittu et al.,^28^ which reported a higher occurrence of *bla*_CTX–M–27_ in human isolates from Finland. This variation could be attributed to the clinical vs population view of the samples. The PCR-based study by Belas et al.,^67^ reported a higher prevalence of the *bla*_CTX–M–15_ gene among humans and their companion animals in Portugal. Adler et al.^68^ noted that ST131-*bla*_CTX–M–27_-*E. coli* has been reported to have a higher transmission rate when compared to the ST131-*bla*_CTX–M–15_-*E. coli* in an Israeli hospital setting. These studies support the notion of a shift in the most dominant strains in human samples, and the emergence of *bla*_CTX–M–27_ as a challenger for *bla*_CTX–M–15_^28,68^. In another study conducted in China, clinical isolates were reported to have an increase in the proportion of *bla*_CTX–M–55_ compared to other *bla*_CTX–M_ genes^58^, further depicting the rapidly evolving epidemiology of these enzymes. Finally, the co-occurrence of *aac*(6’)-Ib-cr5, *bla*_OXA-1_ and *bla*_CTX-M-15_-ST1193 isolates were reported in fluoroquinolone-resistant extra-intestinal pathogenic *E. coli* in Vietnam^69^ and Qatar^70^. Our analysis revealed several point mutations that could confer antimicrobial resistance with the mutation in *par*C, *par*E and *gyr*A genes (conferring quinolone resistance), *uph*T and *pts*I (fosfomycin resistance) and *mar*R (conferring multidrug resistance to tetracycline, cephalosporin, rifamycin, glycyclines, phenicols etc) being the most prevalent and consistent with previous reports^28^.

Only one isolate harboured harboured a colistin resistance gene. This could be because Finland has a very low antibiotic consumption rate^48^. There were only a few reports of clinical colistin resistance in Finland with the index report in 2018^71^ and later in 2023 in hospital wastewater^72^. This could be due to the concept of collateral sensitivity, which occurs when the acquisition of resistance to one antibiotic increases susceptibility to another antibiotic and can be exploited to eliminate AMR selectively^73^. In the same vein, there is only a low burden of *bla*_KPC_ and *bla*_NDM_ genes in sequenced isolates obtained from hospital wastewater^72,74^ and municipal wastewater^26^, but we had earlier detected a high prevalence using PCR in the Viikinmäki WWTP in Helsinki^75^. Metagenomic approaches to urban sewage surveillance revealed that the most abundant ARGs were those conferring resistance to aminoglycosides, macrolides, and beta lactams^3^ with aminoglycoside resistance determinants being the most abundant^76–78^. Chromosomal and plasmid-mediated point mutations conferring AMR are also similar in abundance and diversity to those reported from clinical isolates in Finland^28^.

The majority of wastewater isolates from this study and the human clinical isolates (obtained from Kurittu et al.^28^) harboured plasmid replicons belonging to the IncF family, which have been identified as important carriers of globally successful AMR genes, especially those encoding for ESBLs^79^. Also, the IncF plasmids have been shown to mediate the clonal (within the same lineage) and horizontal (between lineages) transmission of the *bla*_CTX-M-15_^80^. Nearly identical plasmids were recovered from isolates over two years, indicating long-term persistence^80^. However, the pMLST results should be interpreted with caution, since long-read sequencing would allow for more robust and accurate identification of plasmid structures and gene locations. As plasmids are important mediators of AMR worldwide, further plasmid characterization through hybrid sequencing methods is warranted to investigate the epidemiological events in more detail in future studies^28^.

The differences in abundance and diversity of antibiotic and stress resistance genes between different WWTPs could be attributed to the highest incidence of ESBL-producing *E. coli* in the health facilities serving residents of Lappeenranta and Oulu reported in 2021^26,27^. However, there was no association between the presence and relative abundance of plasmid replicons on the diversity of ARGs in wastewater samples from each WWTP. In addition, the presence of IS*26,* an element that has been reported to be highly active in disseminating ARGs by recruiting a gene (s) into the mobile gene pool could have contributed to the high MDR status of wastewater isolates^81^. This element supports their continued dissemination to new locations by creating pseudo-compound transposons (PCTs) and through targeted conservative mechanisms^82^.

The cgMLST of the wastewater isolates revealed only two clusters (two isolates in each cluster), depicting the high genetic diversity and existence of multiple successful lineages of ESBL-producing *E. coli* in circulation in Finland (Fig. 3a). Some of the human clinical ST131 isolates however belonged to the same genomic cluster (Fig. 3b). The lack of genomic clusters between human and wastewater ST131 isolates further highlights the hyperclonal nature of this clade. Despite this, we believe that WBS is sensitive and can be complementary to identifying and monitoring this hypervirulent and globally disseminated clade. In addition, more data are needed for epidemiological inferences to be made from WBS.

As one of the drawbacks of WBS, it is difficult to draw direct comparisons between prevalence in humans and prevalence in wastewater. This could be due to the possibility of the acquisition of resistant genes or mobile genetic elements in the sewerage system, the possibility of pathogens from asymptomatic carriers and companion animals (and the environment through rainwater), and the possibility of sporadic release of ARB, which could lead to its infrequent detection in wastewater samples. In addition, isolates from the national infectious diseases register are not sequenced. Hence, nationwide cgMLST comparison with wastewater isolates was not currently possible. Also, this study did not compare the ARGs detected via in-silico typing with other in-vitro molecular techniques e.g. PCR. Despite these, our findings are invaluable for future monitoring of AMR trends.

In conclusion, WBS of priority AMR pathogens could help public health authorities evaluate the evolution and epidemiology of these pathogens, distinguish specific local drivers and risk factors, and serve as a complementary tool for epidemiological inference. This study provides more genomic insights into the epidemiology of AmpC/ESBL-producing *E. coli* in wastewater and found that the *bla*_CTX–M–15_ was the most prevalent ESBL gene. Our dataset further detected the high prevalence of the ST131 strain with MDR genotypes, and few isolates clustered with human ST131 samples. Longitudinal WGS-based AMR surveillance studies are needed to monitor AMR trends before wastewater can be successfully implemented as an early warning tool for pandemic preparedness.

## Supplementary Information

Supplementary Information 1.

Supplementary Information 2.

Supplementary Information 3.

Supplementary Information 4.

Supplementary Information 5.

Supplementary Information 6.

## Data Availability

The sequence raw reads are available on the European Nucleotide Archive (Project ID: PRJEB78480). The individual accession numbers are provided in the supplementary file 4 and are available from the corresponding author on reasonable request.

## Abbreviations

ARG: Antimicrobial Resistance Genes
AST: Antimicrobial Susceptibility Testing
DDST: Double-Disk Synergy Test
MLST: Multi-Locus Sequence Typing
ESBL: Extended Spectrum Beta Lactamase
MDR: Multi Drug Resistant
CT: Complex type (CT)
ST: Sequence Type
WBS: Wastewater-Based Surveillance
WGS: Whole Genome Sequencing
cgMLST: Core genome Multi-Locus Sequence Typing
tLST: Transmissible Locus of Stress Resistance
