# Supplementary material for: Wastewater based genomic surveillance key to population level monitoring of AmpC/ESBL producing Escherichia coli
Source: Sci Rep. 2025 Mar 3;15:7400. doi: 10.1038/s41598-025-91516-9 (PMC11876440; doi:10.1038/s41598-025-91516-9)
Supplement: Supplementary file 1 — Supplementary Information 1. [file 41598_2025_91516_MOESM1_ESM.docx]

**SUPPLEMENTARY DATA**

**Fig. S1. ESBL-producing *E. coli* sequence types in wastewater treatment plants in Finland.**


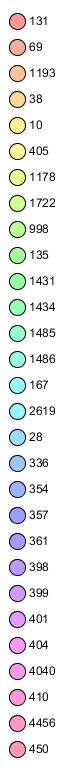

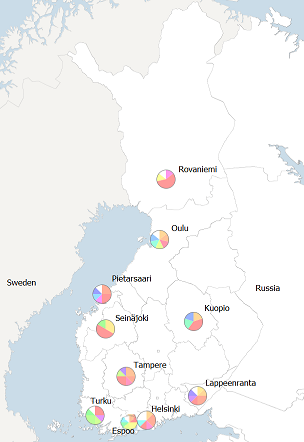


Figure S2. Flowchart for wastewater-based screening for AmpC/ESBL-producing *E. coli* used in this study.

**
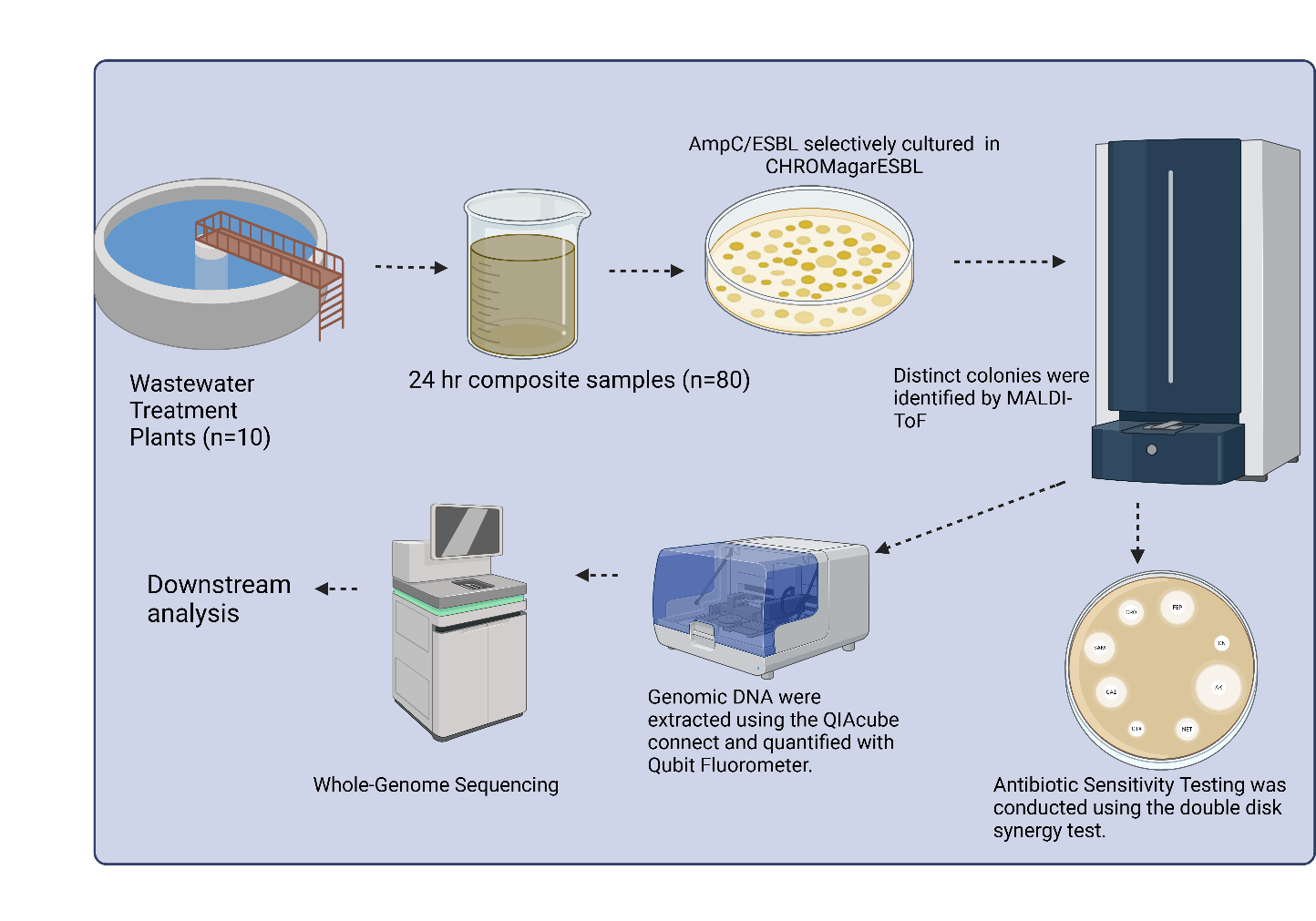
**

Figure S3. A Sankey diagram depecting occurrence of plasmid replicons across WWTPs in Finland.
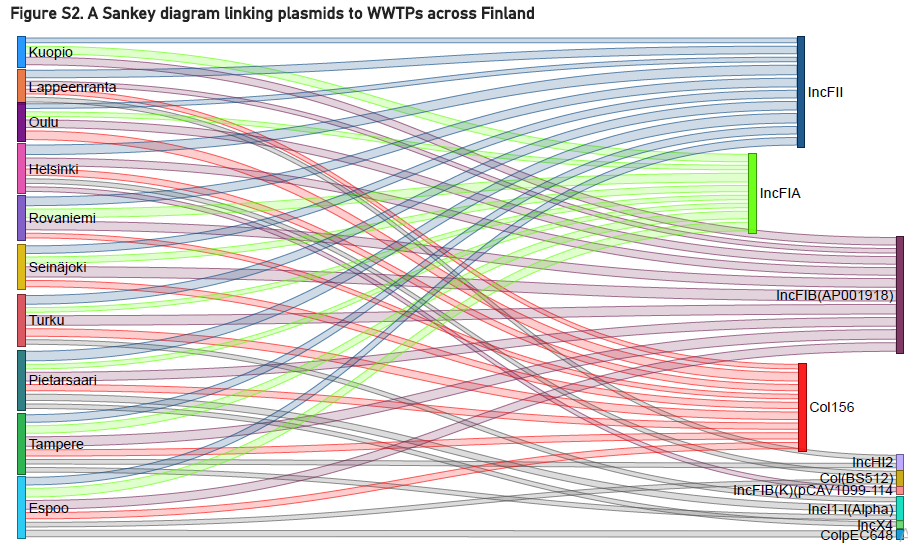


Figure S4. Antibiotic consumption in Finland between 2019 and 2023.

Data were retrieved from the European Center for Disease Prevention and Control [surveillance data on antimicrobial consumption.](https://qap.ecdc.europa.eu/public/extensions/AMC2_Dashboard/AMC2_Dashboard.html#national-country-tab) MLS – Macrolides, Lincosamides, and Stretogramins.

**Supplementary Table S1. Function (Classification) of genes associated with antibiotic resistance.**

| Classification | Group | Antimicrobial Class | Gene | Function (characteristics) |
| --- | --- | --- | --- | --- |
| Disinfectant resistance | Disinfectant resistance gene | Quaternary ammonium compound | *qac*E/*qac*EΔ1 | *qac*E family quaternary ammonium compound efflux SMR transporter |
| Stress resistance | Acid resistance gene | Acid shock protein | *ymg*B | Involved in the regulation of biofilm and acid-resistance, signal input into the two-component signaling pathway. |
|  |  |  | *asr* | Required for growth and/or survival in acidic conditions. |
| Metal resistance | Metal resistance genes | Mercury | *mer*C, *mer*P, *mer*R, *mer*T, | Periplasmic mercury ion-binding protein |
|  |  | Copper | *pco*A, *pco*B, *pco*C, *pco*D, *pco*E, *pco*R, *pco*S | Copper-exporting P-type ATPase |
|  |  | *Sil*ver | *sil*A, *sil*B, *sil*C, *sil*E, *sil*P, *sil*R, *sil*S. | monoatomic cation transmembrane transporter activity |
|  |  | Arsenic | *ars*C, *ars*D, | Arsenite efflux transporter metallochaperone |
|  |  | Tellurium | *ter*D, *ter*W, *ter*Z | Tellurium resistance protein |
| Antibiotic resistance | Efflux pump | Efflux pump membrane transporter | *acr*F | efflux transmembrane transporter activity |
|  |  | Multidrug resistance protein | *mdt*M | Proton-dependent efflux pump for several classes of drugs |
|  |  | Multidrug resistance protein D/E | *emr*D/E | Multidrug resistance pump *par*ticipates in a low energy shock adaptive response. |
|  | Antibiotic resistance genes | Aminoglycosides | *aac*(6')-Ib-cr5, *aac*(3)-IId, *aph*(3')-Ia, *aph*(3')-Ib *ant*(2'')-Ia, *aph*(6)-Id, *aad*A1, *aad*A2, *aad*A5 | Aminoglycoside N(6')-acetyltransferase type 1 and Aminoglycoside 3'-phosphotransferase that confers resistance to aminoglycoside antibiotics. |
|  |  |  | *sat*2 | A plasmid-mediated streptothricin acetyltransferase, which confers resistance to streptothricin |
|  |  | Beta lactam antibiotics | *bla*EC, *bla*EC-5 | Ambler class C beta-lactamase enzyme naturally occurring in *E. coli* |
|  |  |  | *bla*TEM-1 | Ambler class A broad spectrum beta-lactamase enzyme |
|  |  |  | *bla*OXA-1 | Ambler class D broad spectrum beta-lactamase enzyme |
|  |  |  | *bla*DHA-1 | Ambler class C beta lactamase enzyme |
|  |  |  | *bla*SHV-1 | Ambler Class A broad spectrum beta-lactamase enzyme |
|  |  |  | *bla*CTX-M-14  *bla*CTX-M-15  *bla*CTX-M-24  *bla*CTX-M-27  *bla*CTX-M-55 | Ambler class A extended spectrum beta-lactamase enzymes |
|  |  | Polymixin | *mcr*-1.1 | This gene catalyzes the addition of a phosphoethanolamine moiety to lipid A, thereby conferring resistance to polymyxins. |
|  |  | Macrolides | *erm*(B), *mph*(A) | This gene dimethylates a single adenine in 23S rRNA, which causes high-level macrolide resistance. |
|  |  | Quinolones | *qnr*B4, *qnr*S1 | Plasmid-mediated quinolone resistance genes |
|  |  | Rifamycin | *arr, arr-2* | Integron-encoded ADP-ribosyltransferase that confers resistance to rifamycin *ant*ibiotics. |
|  |  | *Sul*fonamides | *sul*1, *sul*2, *sul*3 | These genes are associated with dihydropteroate synthase, conferring resistance to *sul*fonamides. |
|  |  | Tetracyclines | *tet*(A), *tet*(B) | Responsible for the energy-dependent process of active tetracycline efflux from the cell. |
|  |  | Aminopyrimidine antibiotic | *dfr*A1, *dfr*A12, *dfr*A14, *dfr*A14 | A dihydrofolate reductase, re*sul*ting in trimethoprim resistance. |
| Point mutations | *Ant*ibiotic resistance mutations | Quinolone antibiotics | *gyr*A_D87N | A point mutation in the *gyr*A region, resulting in quinolone resistance |
|  |  |  | *gyr*A_S83L |  |
|  |  |  | *par*C_E84V | A point mutation in the *par*C region, resulting in quinolone resistance |
|  |  |  | *par*C_S80I |  |
|  |  |  | *par*E_L416F | A point mutation in the *par*E region, resulting in quinolone resistance |
|  |  |  | *par*E_I529L |  |
|  |  | Fosfomycin | *pts*I_V25I | These genes are involved in cyclic AMP synthesis and confer resistance to fosfomycin by affecting the regulation of its import. |
|  |  |  | *uhp*T_E350Q |  |
|  |  | Multiple antibiotic classes | *mar*R_S3N | A point mutation in the *mar*R gene, re*sul*ting in repression of the mar operon marRAB, thus activating of multidrug efflux pump conferring resistance to tetracyclines, cephalosporins, rifamycin, fluoroquinolones, glycyclines, and phenicols. |
